# GMOs in animal agriculture: time to consider both costs and benefits in regulatory evaluations

**DOI:** 10.1186/2049-1891-4-37

**Published:** 2013-09-25

**Authors:** Alison L Van Eenennaam

**Affiliations:** 1 Department of Animal Science, 2113 Meyer Hall, University of California, One Shields Avenue, Davis, CA 95616, USA

**Keywords:** Cost: benefit analysis, Genetic engineering, GMO, Regulation, Risk assessment, Safety

## Abstract

In 2012, genetically engineered (GE) crops were grown by 17.3 million farmers on over 170 million hectares. Over 70% of harvested GE biomass is fed to food producing animals, making them the major consumers of GE crops for the past 15 plus years. Prior to commercialization, GE crops go through an extensive regulatory evaluation. Over one hundred regulatory submissions have shown compositional equivalence, and comparable levels of safety, between GE crops and their conventional counterparts. One component of regulatory compliance is whole GE food/feed animal feeding studies. Both regulatory studies and independent peer-reviewed studies have shown that GE crops can be safely used in animal feed, and rDNA fragments have never been detected in products (e.g. milk, meat, eggs) derived from animals that consumed GE feed. Despite the fact that the scientific weight of evidence from these hundreds of studies have not revealed unique risks associated with GE feed, some groups are calling for more animal feeding studies, including long-term rodent studies and studies in target livestock species for the approval of GE crops. It is an opportune time to review the results of such studies as have been done to date to evaluate the value of the additional information obtained. Requiring long-term and target animal feeding studies would sharply increase regulatory compliance costs and prolong the regulatory process associated with the commercialization of GE crops. Such costs may impede the development of feed crops with enhanced nutritional characteristics and durability, particularly in the local varieties in small and poor developing countries. More generally it is time for regulatory evaluations to more explicitly consider both the reasonable and unique risks and benefits associated with the use of both GE plants and animals in agricultural systems, and weigh them against those associated with existing systems, and those of regulatory inaction. This would represent a shift away from a GE evaluation process that currently focuses only on risk assessment and identifying ever diminishing marginal hazards, to a regulatory approach that more objectively evaluates and communicates the likely impact of approving a new GE plant or animal on agricultural production systems.

## Introduction

**Figure 1:**
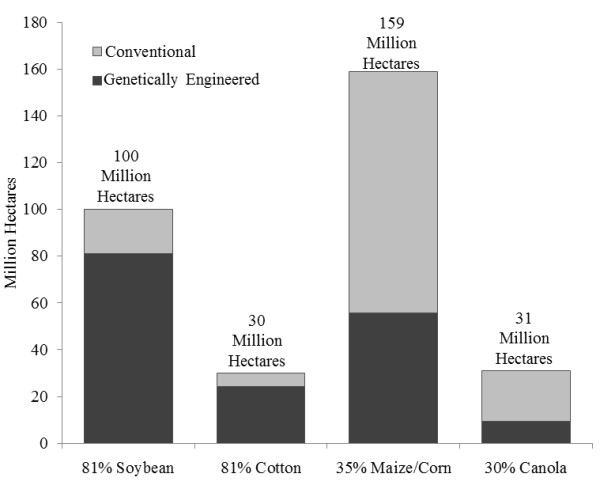
**Global adoption rates (%) for principal GE crops in 2012 [**[1]**].** Figure used with permission.

A high proportion of soybean (81%), cotton (81%), corn (35%), and canola (30%) crops grown globally are genetically engineered (GE) varieties (Figure 1) [1]. It has been estimated that over 70-90% of harvested GE biomass is fed to food producing animals [2], making the world’s livestock populations the largest consumers of the current generation of GE crops. Crops that are produced using GE are likely to become even more important to animal agriculture as the global livestock population grows in response to increased demand for animal protein products. Prior to commercialization, GE crops must go through an extensive safety evaluation. The Organisation for Economic Co-operation and Development (OECD) has established safety assessment processes based on the principle of “substantial equivalence” to assure that foods derived from GE crops are as safe and nutritious as those from plants derived through conventional breeding [3]. The concept is based on the principle that “*if a new food is found to be substantially equivalent in composition and nutritional characteristics to an existing food, it can be regarded as being as safe as the conventional food”*[4]. For GE crops, this comparison entails an extensive chemical analysis of key macronutrients, micronutrients, antinutrients and toxins. Most conventionally-bred crops that are on the market have not ever been tested for their safety in animals, but they are known to be safe based on their history of safe use. Likewise, few foods have been subject to toxicological testing.

Inclusion in a balanced diet has often been a component of the regulatory approval package for a new GE crop. These studies are typically performed with rodents for a duration of 90 d. Although this is technically only required when the composition of the GE plant has been modified substantially, or there are indications for the potential occurrence of unintended effects [5], some are calling for an increase in the number, length, and target species (i.e. livestock) involved in animal feeding studies required for the regulatory approval of GE crops.

On June 2013, the European Commission published a Regulation (EU) No 503/2013 requiring an obligatory 90-d whole food/feed rodent feeding study for regulatory approval of each GE crop event [6]. Depending on the outcome of this study, a 2-year long-term GE feeding study in rats may also be requested, on a case-by-case basis. This Regulation passed despite the fact that the European Food Safety Authority (EFSA) questioned the need to provide such studies for the risk evaluation of each GE crop application as follows: “*When 'molecular, compositional phenotypic, agronomic and other analyses have demonstrated equivalence of the GM food/feed, animal feeding trials do not add to the safety assessment’*” [5]. There are considerable costs involved in performing animal feeding studies. In 2007 it was estimated that the range of costs involved with animal performance and safety studies (typically a 90-d whole food/feed rodent feeding study) for approval of a GE crop ranged from $USD 300,000–845,000 [7]. Presumably costs have increased since that time, and if longer studies are required costs would likewise be increased. A recent two-year rat feeding study involving 200 rats was purported to cost 3.2 million Euro (~ $USD 4 million) [8]. Calls to do long-term or mutigenerational GE feeding studies on long-lived target species such as cattle would be orders of magnitude more expensive; assuming sufficient feed from the GE crop and its isogenic comparator was available to perform such work. Additionally the cost of rendering the animals would need to be factored into regulatory evaluations as animals would not be able to enter the food supply if fed an as yet unapproved GE crop variety.

The purpose of this paper is to review the rationale and results of peer-reviewed animal feeding studies using GE crops. Results from short-term and long-term studies are evaluated to determine if additional information was identified in these long-term studies that would not have been picked up in the short-term study, and the details of some highly controversial studies are reviewed. It is suggested that animal feeding studies should only be required if there is some reasonable food safety concern indicated during the regulatory evaluation of GE crops that has not been adequately addressed by *in silico* and *in vitro* analyses. Further, the need to evaluate both the risks and benefits in regulatory evaluations is discussed given the weight of scientific evidence on the safety and performance of GE crops that have been commercialized to date.

### Short-term rodent feeding studies

The protocols for 90-d rodent studies were adapted from those for *in vivo* toxicological studies [9] and are intended to assess feed safety. This protocol recommends 10 animals per sex and per group, with three doses of the test substance and a control group. It was developed to test the toxicology of a chemically defined molecule (e.g. a drug), not complex materials like GE feed. It becomes somewhat problematic to appropriately “dose” the GE feed because diets must be balanced to meet the nutritional requirements of the rodents. Too much of a single crop or species in the diet may result in deleterious nutritional effects and associated phenotypes, independent of the GE status of the crop. GE feeding studies typically incorporate 33% GE animal feed in the test diet. Ideally, the GE line is compared to its near isogenic counterpart grown in the same location and environment, and possibly also a non-GE line (conventional comparator) considered to be safe. The latter is included to estimate the natural variability of analytes seen within the crop species. Several studies have revealed that environmental factors (such as field location, planting, sampling time, crop management practices), and genetic factors like line/breed and mutagenesis can result in more variability in gene expression between samples than is observed resulting from GE [10-12]. The failure of many researchers to appropriately match their experimental GE diets to appropriate isogenic and nutritionally equivalent control diets has resulted in some of the most controversial, and highly criticized, GE feed safety studies.

### Long-term and multigenerational animal feeding studies

Ninety-day rodent toxicology feeding studies are not designed to measure effects on reproduction or development. Likewise, they are not designed to detect long term effects in animals, or the effect that eating a GE-based diet has on the next generation. This has resulted in a call for more long term and multigenerational animal feeding studies. Although, it should be noted that analyses of available data indicate that, for a wide range of substances, reproductive and developmental effects are not potentially more sensitive endpoints than those examined in subchronic toxicity tests [13]. Several review papers that summarize the results of long-term and multigenerational feeding studies in a variety of species have been published recently [2,14-16]. The duration of published long-term feeding studies using a GE-based diet ranged from 110 d [17-19] to 728 d [20]. The longest multigenerational study involved ten generations of quail fed up to 50% GE corn [21].

In a comprehensive review of the health effects of GE plants, Snell *et al.*[15] focused on 12 long-term and 12 multigenerational feeding trials with GE crops that also had a 90-d rodent study GE feeding study comparator [22-29]. It is important to note that these studies were financially supported by public funds. The question they specifically asked was, “Do long-term and multigenerational GE feeding studies provide any new evidence indicative of some adverse effect(s) that were not previously identified in the 90-d rat study”? The authors concluded that while none of the long-term or multigenerational studies they evaluated revealed any new effect that had not been found in the 90-d rodent toxicology study, there was a need to develop reproducible and standardized protocols for conducting and analyzing complementary fundamental research using different animal models on long-term and multigenerational studies. Some of the long-term and multigenerational studies examined did not use isogenic lines as controls, and the organs and parameters that were measured varied greatly among the studies. Few studies have been conducted using the same GE line and species, and even when they were conducted in the same species, different parameters were measured making a meta-analysis of the data problematic. The authors suggested that while a more standardized protocol for long-term and multigenerational studies would be useful for exploratory fundamental research projects, such studies should be conducted on a case-by-case basis for GE food safety only if some reasonable doubt remained after a 90-d rodent feeding trial.

Another review examined 60 high-throughput “-omics” comparisons between GE and non-GE crop lines, including 17 long-term and 16 multigenerational animal feeding studies, to determine if these additional tests raised new safety concerns [14]. High-throughput “-omics” – transcriptomics, proteomics, and metabolomics - methods have been suggested as a nontargeted approach to detect unintended effects in GE plants. Long-term studies included rats [20,30-32], mice [33-37], salmon [38,39], beef cattle [40], dairy cows [41], macaques [42], pigs [19], and quail [43]. Multigenerational studies included rats [44-48], mice [49-53], pigs [54-56], bulls [56], dairy cows [56], goats [57], sheep [56,58], broilers [56,59], laying hens [56,60], and quail [21,61]. These powerful studies consistently revealed that GE had fewer unintended effects than conventional breeding techniques. The authors suggested that the small number of unintended effects observed, including changes in the level of lactate dehydrogenase enzyme in goats fed GE soybean [57], and immune responses in mice fed GE triticale in the fifth generation of mice [49], fell within the normal range of variation, and did not suggest that they represented a health hazard. Even when GE crops were designed to intentionally have altered metabolic traits, “-omics” expression profiling technologies revealed few unintended effects [14]. The authors concluded that “*none of the“-omics” comparisons has raised new safety concerns about (marketed) GE varieties; neither did the long-term and multigenerational studies on animals*”. They further proposed that the data collected to date suggest that the risk assessment should actually be lowered for GE crops.

A highly controversial study by Séralini claimed that feeding GE glyphosate tolerant corn and a related herbicide formulation over a two year period caused organ damage, tumors, and early death among Sprague–Dawley rats [8]. The authors used a 90-d rodent toxicology feeding study design to study long-term carcinogenicity while failing to consider that 2-yr old rats of the Sprague–Dawley strain are known to be highly susceptible to developing tumors [62]. Independent scientists have noted numerous design flaws in the Séralini study [63,64] including too few animals per treatment group, too few controls (20 control animals (10 male and 10 female) versus 180 “treated” animals), inappropriate histological and statistical analysis of mortality and tumor rates, and ignoring the fact that many other peer-reviewed long-term studies with contradictory results have been conducted by independent scientists from around the world. This includes a two-yr rat feeding study, funded by the Japanese government, which found no deleterious effects of feeding GE feed in their long-term feeding trial [20]. In that study the researchers followed the suggested experimental design for a 104 wk carcinogenicity study [65,66] which includes the use of 50 animals per treatment group, use of a rat strain that has an acceptable survival rate for the long-term study, and appropriate statistical analysis of their data. The highly-publicized but poorly-executed Séralini investigation has since been thoroughly debunked by regulatory agencies throughout the world [67-75].

Another infamous study conducted by Ewen and Pusztai [76] in 1999 reported several injurious effects in the gastrointestinal tract of rats that had been fed GE potatoes expressing the antinutritive lectin *Galanthus nivalis* agglutinin (GNA), a compound with insecticide activity. It was claimed that the consumption of GE potatoes had significant effects on the immune system of rats in the feeding trials, because of some effect of GE itself rather than because of the particular gene inserted. However a report by the Royal Society concluded that the data reviewed “*provide no reliable or convincing evidence of adverse (or beneficial) effects, either of lectins added to unmodified potatoes or of potatoes genetically modified to contain a lectin gene, on the growth of rats or on their immunological function*” [77]. That report criticized the rat feeding study for the common trial design errors of too few animals per diet group and the lack of controls such as a standard rodent diet containing about 15% protein (the test diet was severely protein deficient at ~ 6%) [77]. Irrespective, given that lectin has been widely documented to be toxic and/or allergenic, GE crops expressing such a substance would be highly unlikely to ever obtain regulatory approval [78].

A critical review of the other published studies where change(s) in some parameters are reported to result from GE feed also reveal deviations from standard protocols [14,15,79]. These include control feed that was not derived from near isogenic lines, insufficient animal numbers for statistical power, over interpretation of differences which lie within the normal range of variation and hence are not biologically significant, and/or poor toxicological interpretation of the data. This emphasizes the need to follow required standard protocols in animal feeding studies. This lack of compliance with international protocols by some research groups, and the highly sensational presentation of their results in public settings have led to the unfortunate situation where companies are reticent to provide plant material for independent feeding studies [15]. This is particularly problematic for researchers who are interested in pursuing feeding trials in livestock species which typically require larger amounts of GE feed.

### Animal reproduction

The reproductive effects of GE crops are another area that has generated debate [16]. In this regard several controversial studies are often cited. Some of these studies were not published in the peer-reviewed literature but rather were posted only on the internet and publicized at press conferences [80,81]. The Ermakova study [80] claimed that transgenic soybeans compromised the fertility of rats and dramatically decreased the survival and growth of their offspring. However the study was criticized for numerous design flaws by academic scientists [82]. The other internet study [81] housed male and female mice as breeding pairs for approximately 20 wk during which time they were allowed to produce litters continuously. The authors identified differences in reproductive parameters between mice fed with GE maize and the controls. They reported that there were statistically significantly fewer pups born in the GE group in the 3^rd^ and 4^th^ litters, and that there were fewer pups weaned in the 4^th^ litter compared with the control group. The study was withdrawn from the internet by Austrian officials because of weaknesses in experimental design, calculation errors and deficiencies in the statistical analysis [83].

The fact that studies which did not even reach the accepted standard of peer-review publication can receive such wide publicity and be uncritically cited as evidence of the risks of GE crops by some authors [84] is unfortunate given a large number of less controversial, and hence less famous, carefully controlled peer-review studies that revealed no negative effects of GE-feed on various attributes (e.g. gonad weight, fecundity, fertility, gonadal histopathology) of female [23-26,46,47,85-96] and male [23-26,46,47,50,51,85,87,89-96] reproduction in animal feeding studies.

Another study examined the ultrastructural and immunocytochemical features of preimplanation embryos from 10 two-mo old mice fed a standard diet containing 14% GE soybean or non-GE soybean until weaning [97]. Morphological observations revealed that the embryo nuclear components were similar in the two experimental groups, but pre-mRNA maturation seemed to be less efficient in the embryos from GE-fed mice than controls. Again, this study did not provide any information on the source of the GE soy or the control, nutritional composition of the diet, and the number of female mice per group (n=5) was small. Non-adherence to standard procedures makes data interpretation difficult as it is not clear which of the multiple variables that differed between the groups were causative of the observed differences. Research published between 2002 and 2005 by researchers in Italy indicating ultrastructural changes in organs in the liver, pancreas and testes of mice fed diets supplemented with GE and non-GE soya [36,37] has likewise been criticized by independent scientific groups [98,99] regarding a lack of information concerning the source of the GE soybean, the appropriateness of the control soybean used in the diet, and the nutritional composition of the diet.

Clearly these repeated experimental design flaws in animal feeding studies evaluating GE feed are exacerbating the continued controversy associated with the safety of GE food and feed that currently divides not only the general public but frequently also the scientific community. Animal scientists have an obligation to ensure that feeding studies using GE crops are carried out according to standard protocols [5,13,65,100-102] (Table 1) to ensure data can be appropriately analyzed and unambiguously interpreted in the absence of confounding factors.

**Table 1:** **Recommendations for the conduct of animal studies to evaluate GE crops**[101]

**Animals (species/categories)**	**Number of animals (coefficient of variation 4 to 5%)**	**Duration of experiments**	**Composition of diets**	**Measurements/endpoints**
Poultry for meat production	10-12 pens per treatment with 9–12 birds per pen	5 wk or more	Balanced diets	Feed intake, gain, feed conversion, metabolic parameters, body composition
Poultry for egg production	12-15 replications per treatment with 3–5 layers per pen	18-40 wk of age, at least three 28-d phases	Balanced diets	Feed intake, egg production, feed conversion, egg quality
Swine	6-9 replications per treatment with 4 or more pigs per replication	Piglets (7–12 kg) 4–6 wk Growers (15–25 kg) 6–8 wk	Balanced diets	Feed intake, gain, feed conversion, metabolic parameters, carcass quality
Growing and finishing ruminants	6-10 replications per treatment with 6 or more cattle per replication	90-120 d	Balanced diets	Feed intake, grain, feed conversion, carcass data, metabolic parameters
Lactating dairy cows	12-16 cows per treatment 28 cows per treatment	Latin square 28 d periods Randomized block design	Balanced diets	Feed intake, milk performance and composition, body weight, body condition score (BCS), cell counts in milk, animal health

Table used with permission [2].

### Statistical analysis and experimental design

The scientific question being addressed by feeding studies should be well-established before designing the study [79]. Designing experiments to test for intended effects is relatively straightforward. Sample size determinations are based on the size of the effect that is considered important and the required power (i.e. probability that the test will reject the null hypothesis when the alternative hypothesis is true) for a given significance level. Statistical power increases with the sample size, if all other parameters of statistical testing are held constant.

Animal feeding trials are sometimes also used to identify “unintended” effects. These are effects or results that were not expected nor considered in the experimental design and sample size calculations. If many independent tests are performed on the same sample, the probability of obtaining significant results will increase merely due to the multiple comparisons being performed. For example, when many parameters are measured it is likely just by chance one in 20 will rise to the level of statistical significance (assuming *P *< 0.05) [103]. The correct statistical methods should be used to analyze for the statistical significance of multiple comparisons. Two common methods, Bonferroni adjustment and the False Discovery Rate, are among approaches used to take multiple comparison issues into account. The false discovery rate (FDR) is a technique specifically developed for controlling the expected proportion of falsely rejected hypothesis [104]. The use of FDR or similar techniques allows this control and improves the probability of discriminating statistical differences from those generated by random chance.

It is also important to understand the biological relevance of statistically significant differences that might occur between treatment groups. The European Food Safety Authority (EFSA) clarified the difference between statistical significance and biological relevance [103]. Statistical significance is a term that has a specific and distinctive meaning when used in the context of statistical hypothesis testing. Significant does not necessarily mean “important” or “meaningful” but rather is a statistical statement on the property and information content of the observed data. Biological relevance, on the other hand, *“implies a biological effect of interest that is considered important based on expert judgment. Its use refers to an effect of interest or to the size of an effect that is considered important and biologically meaningful and which, in risk assessment, may have consequences for human health. The objective of carrying out an empirical study is usually to identify the existence of relevant biological effects at the population level using statistical tools to detect them. Therefore the identification of statistical significance is only part of the evaluation of the biological relevance”*[103]. Importantly it is stressed that the “*nature and size of biological changes or differences seen in studies that would be considered relevant should be defined before studies are initiated”* rather than be derived from a post-hoc analysis of the data*.* This enables the design of experiments with sufficient statistical power to be able to detect such biologically relevant effects of this size if they truly occurred.

Ignoring this distinction is a frequent criticism of studies where a statistically significant treatment effect is found in a post-hoc analysis of a data set with a small sample size and spurious conclusions regarding the biological relevance of the finding to health are inferred. This distinction is especially relevant in the absence of knowledge regarding the normal level of biological variation that may exist between different non-GE cultivars and varieties. Many constituents in crop plants vary widely due to environmental factors (such as field location, planting, sampling time, crop management practices), and genotype and this natural variation is not typically considered to be a food safety concern. For the purposes of a safety assessment, the question is not whether a GE line has a statistically different level of some constituent from its near-isogenic nontransgenic comparator, but rather whether differences are biologically relevant to health according to expert judgment.

### Feeding trials in target species

Target animal (food producing animals such as ruminants, pigs, poultry, and fish) feeding studies have not been required for regulatory approval in part because first generation GE crops have proven to be substantially equivalent to their conventional counterpart. Over the past 20 yr, the U.S. Food and Drug Administration (FDA) has found that all 148 transgenic events they have evaluated, and that includes all of the GE crops that have ever been commercialized, were substantially equivalent to their conventional counterparts [105]. These studies have spanned GE corn, soybean, cotton, canola, wheat, potato, alfalfa, rice, papaya, tomato, cabbage, pepper, raspberry and mushroom, and included traits of herbicide, drought and cold tolerance, insect and virus resistance, nutrient enhancement, and expression of protease inhibitors.

Studies with target animals conducted to date have typically been conducted to evaluate nutritional and feed equivalency of GE, rather than to evaluate safety. Flachowsky *et al*. [2] summarized the results of well over 100 studies feeding target animals (dairy cattle (12), beef cattle (14), other ruminants (10), pigs (21), broilers (48), laying hens (12), other poultry (1), others (fish, rabbits, etc.) (8)) with GE feed from various review documents [78,106]. They concluded that there is good agreement from these studies that feed from GE crops did not significantly influence feed digestibility, animal health, biologically relevant effects on animal performance, composition of animal products, or result in unintended effects (with the exception that lower mycotoxin concentrations have repeatedly been found in *Bacillus thuringiensis* toxin-expressing GE crops [107]) when compared to animals fed isogenic non-GE varieties [2].

An important consideration in target species feeding trials is the substantial costs involved in large animal feeding trials. This is especially true when contemplating long-term or mutigenerational studies on long-lived animals (Table 2). Therefore, long term studies and multigenerational experiments with target animals to date are rather rare [15]. As discussed earlier, many of these long-term studies have not adhered to standard protocols, underlying the vital need for careful consideration of experimental design given the length of time needed and expenses associated with target animal feeding studies. Increasing the number of animals, the length of the trial, and the number of generations are all associated with increased costs. High costs may prevent the public sector from conducting such studies. Long-term, multigenerational and/or target animal feeding studies should be considered and designed to address biologically-relevant questions of second generation GE crop that cannot or have not been answered using *in silico* and *in vitro* methods, or a 90-d rodent feeding study. They should be hypothesis-driven based on the novel traits and/or phenotype associated with the gene/crop combination.

**Table 2:** **Examples of lifespans for growing/fattening animals, in days**[79]

**Animal species/categories**	**Conventional/more intensive**	**Organic/more extensive**
Chickens for fattening (broilers)	3-42	56-84
Turkeys for fattening	56-168	70-112
Growing/fattening pigs	150-300	200-400
Veal calves	80-200	-
Growing/fattening bulls	300-500	400-600

Laying hens and dairy cattle are usually used for longer periods:

Laying hens: about 126–140 d for growing (pullets); about 300–360 d (one year) for the laying period.

Dairy cattle: about 22–36 mo for growing (heifers); one to ten yrs for lactation (average in Europe two to five lactations).

There are some other practical considerations that dramatically increase the cost associated with feeding target livestock with an “as yet unapproved” GE crop. First researchers would need to obtain sufficient GE crop material and an isogenic comparator for the feeding study. Consider a 2-yr feeding study in dairy cattle involving a total of 100 animals; 50 per treatment group. Milk and meat from the cows eating the unapproved GE feed would not be able to enter the food chain and assuming a double blind study design the opportunity cost of that alone would likely be (100 cows × [$USD 5,000/year for milk × 2 yr + $800 cull cow]) in excess of $USD 1,000,000. Housing and bedding for 100 cows at $300/head/mo would be ~$USD 720,000, and then there would be the costs of sample analysis, which conservatively might add another $USD 500,000 depending upon what analytes or endpoints were examined. The cost of such a study would easily exceed $USD 2,000,000. In the absence of an unaddressed safety concern, this expense is not justified given that GE food/feed animal feeding trials of substantially equivalent GE crops that have been carried out to date have not been found to add to the safety assessment, and this also avoids unnecessary animal experimentation.

**Figure 2:**
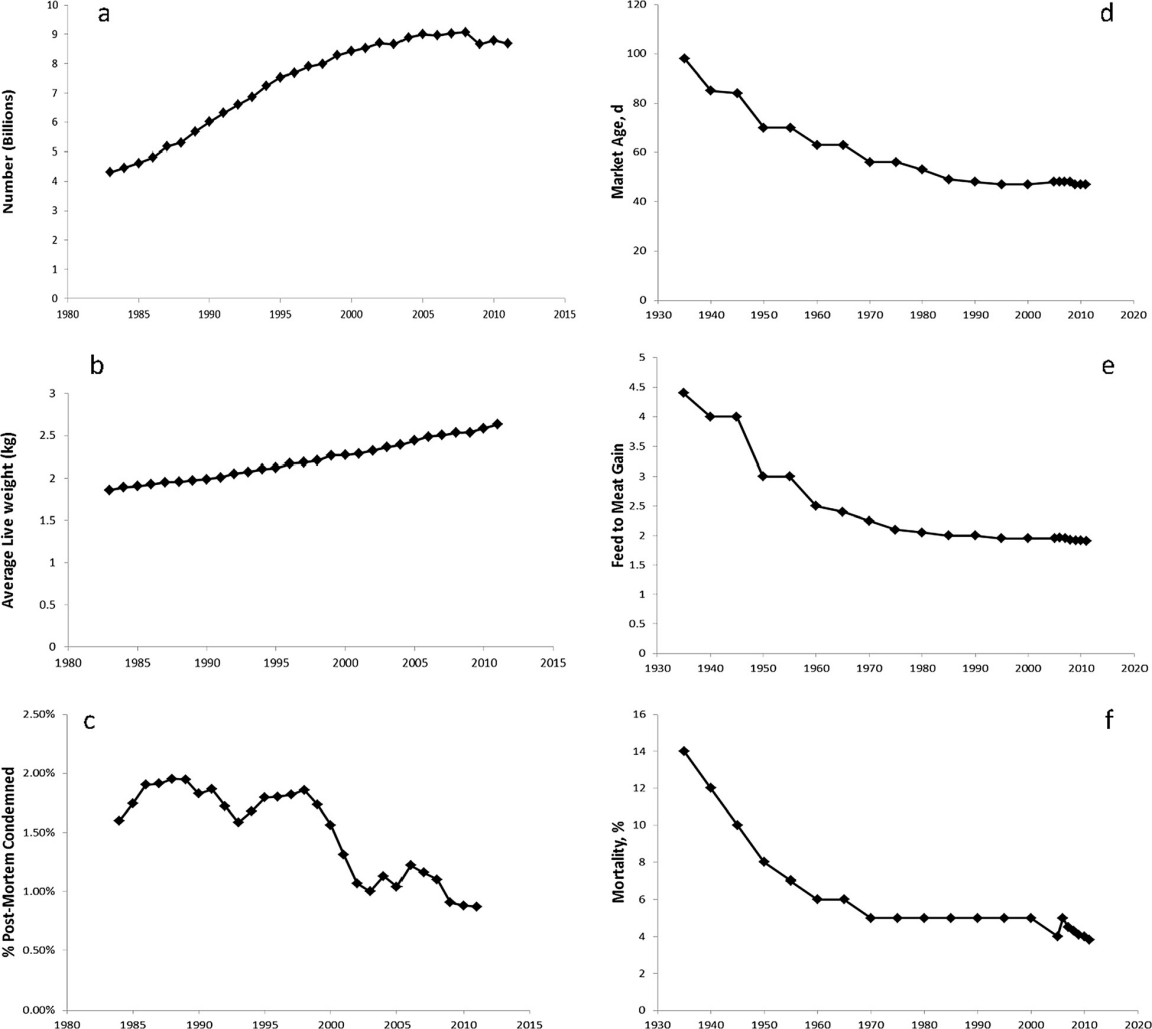
**Summary statics of United States commercial broiler data. a**) Number of chickens processed; **b**) Average weight of chickens; **c**) Percent of chickens condemned by USDA at inspection; **d**) Average d to market; **e**) Efficiency of feed utilization (kg of feed required for one kg of live weight gain; and **f**) Percent mortality. Data from USDA Economics, Statistics and Market Information System (ESMIS). (http://usda.mannlib.cornell.edu/MannUsda/viewDocumentInfo.do?documentID=1497), and the National Chicken Council, Washington DC. (http://www.nationalchickencouncil.org/about-the-industry/statistics/u-s-broiler-performance/).

It should also be noted that although comparatively few feeding trials of commercialized GE crops in target livestock are in the peer-reviewed literature, large numbers of livestock in many countries have been consuming GE feed for over a decade. For example, in 2011 alone approximately 9 billion broiler chickens, weighing over 22.5 billion kg liveweight were produced in the United States. During that year 30 million tonnes of corn and 13.6 million tonnes of soy were used as broiler and breeder poultry feed of which 88% and 94%, respectively, was likely from GE crops. Production parameters, mortality and condemnation rates for the more than 105 billion broilers that have been processed in the US since 2000 are shown in Figure 2. In 2000 approximately 25% of corn and 50% of soy grown in the US was GE and hence poultry diets have likely contained an ever increasing proportion of GE feed from 2000 to 2011. This very large field data set does not reveal overt health problems associated with the consumption of GE feed, but rather shows a continuation of industry trends that were observed prior to the introduction of GE crops (Figure 2).

### Second generation crops

The second generation of GE crops, i.e. those with intentionally changed composition or output traits [102] is likely to include crops with more nutrients or less undesirable substances specifically targeted for animal feed. Other second generation target traits include plants with increased resistance to biotic and abiotic stressors such as drought and saline soils and crops that are more efficient in using limited natural resources to help address the larger challenge of improving global food security [2]. Second generation GE crops will by definition not be “substantially equivalent”. Whether this represents a safety concern will depend on the trait. One study explored the use of the current safety evaluation criteria on a quality-improved GE potato and concluded that the safety of the second generation crops can be properly assessed using the existing current comparative safety assessment methodology [108]. Standard protocols outlining best practices for the conduct of animal studies to evaluate crops genetically modified for output traits have been developed [102].

Animal feeding studies may be needed to assess the bioavailability and/or digestibility of nutrients, and the efficacy of nutrient uptake from second generation GE crops [79,109]. It should be noted that target animal feeding studies to measure these parameters are not required prior to the release of new crop cultivars developed by traditional breeding, although they may be voluntarily conducted by developers to gauge animal performance on the new variety. It is difficult to scientifically justify why the process of GE should be the trigger for a target species feeding evaluation of such crops, rather than a product-based approach triggered by the novel attributes (e.g. increased oil content, decreased lignin) of the modified crop. A high oil crop produced using other plant breeding approaches (e.g. radiation mutagenesis which is known to alter gene expression patterns more than GE [13]), would be logically accompanied by the same bioavailability, animal digestibility and safety questions as a second generation GE crop with the same phenotype.

### Animal preference studies

Some groups have claimed that, given a choice, animals prefer not to consume GE crops. The data to support this assertion are typically anecdotal. There are few studies in the peer-reviewed literature addressing this topic. One study evaluated beef steers grazing preferences for GE and non-GE corn residue. Sixteen steers were grazed on one pasture containing both GE and non-GE corn residue. Their grazing distribution was recorded for 50 d. There was no significant difference in the grazing preference of the animals [110]. In another study using a second generation GE potato, both mice and humans actually showed a preference for the aroma of a GE nonbrowning potato as compared to non-GE potatoes [111]. This effect was not observed when the potatoes were fresh, it was only seen 24 h after the potatoes had aged, presumably associated with the fact that the non-GE potatoes had oxidized and turned brown by that time. Several other studies have started to look at sensory analysis of second generation GE crops [112-114]. In one study, inclusion of GE tomatoes with improved antioxidant properties in the diet of cancer-susceptible mice (p53-knockout mice) significantly extended their lifespan when compared with mice fed standard diets or diets supplemented with non-GE tomatoes [115].

### Fate of recombinant (rDNA) and protein from GE crops

A number of studies have been conducted to look for the presence of rDNA or the protein encoded by the rDNA construct in the milk, meat and eggs from animals fed GE feed [116-120]. To date GE DNA and expression protein products have not been detected in animal protein products derived from food animals fed GE feed. The reason that scientists are researching this topic, even though the presence of DNA and protein from conventional crops in the diet of food animal has not been considered to be problematic, is that consumers are allegedly concerned that GE DNA could alter animal health and in turn eventually pose a threat to human health [121]. The scientific merit of this perception is dubious given that animals and people eat foreign DNA from various crop species every day, and DNA is generally recognized as safe whether it is derived from a GE or conventionally-bred organism.

Some studies have reported finding traces of high copy number plant nuclear and chloroplast DNA sequences in animal organs and tissues [116]. The biological importance of this finding is uncertain. To date there is no evidence that eating DNA and proteins from another species, GE or conventional, results in incorporation of food-based DNA into the genome of the consumer. No country to date has mandated the labeling of products from animals fed with GE plants [121], although voluntary market-driven approaches have resulted in some retailers offering this as a choice for their customers. It is likely to be economically if not technically impossible to use analytical procedures to determine if milk, meat or eggs are derived from animals fed with GE feed [121], and so such products will have to be sourced by supply chain management and verified by documentation.

### Global adoption of GE crops and use in animal agriculture

There is a growing demand for meat and milk as the world population climbs towards 9 billion people and the income of consumers in developing countries rises, and correspondingly there is a growing demand for animal feed. Current crop yields will need to approximately double to meet the feed demands of 2050, and in the absence of newly arable land this demand will necessitate higher yielding crop varieties. Since its introduction in the mid-1990s GE technology has added an additional 110 million tonnes of soybeans and 195 million tonnes of corn, to the global production of these crops. Net level farm economic benefits resulting from GE during that 15 year period were valued at $USD 98.2 billion [122].

In 2012 about 170 million hectares of GE-plant crops (12% of total arable land) were cultivated worldwide [1]. This is a 100-fold increase from the 1.7 million hectares that were planted in 1996, making GE the fastest adopted crop technology in recent history. During the period 1996–2011 it has been estimated that the cumulative economic benefits from cost savings and added income derived from planting GE crops was $USD 49.6 billion in developing countries and $USD 48.6 billion in industrial countries [122]. Of the 17.3 million farmers who planted GE crops in 2012, 15 million were small resource-poor farmers in 20 developing countries. Approximately 14.4 million small farmers in China (4 million ha; mostly cotton, although papaya, poplar, tomato and sweet pepper have all had production approvals), and India (10.8 million ha cotton) collectively planted a record 14.8 million hectares of GE crops in 2012 [1].

**Figure 3:**
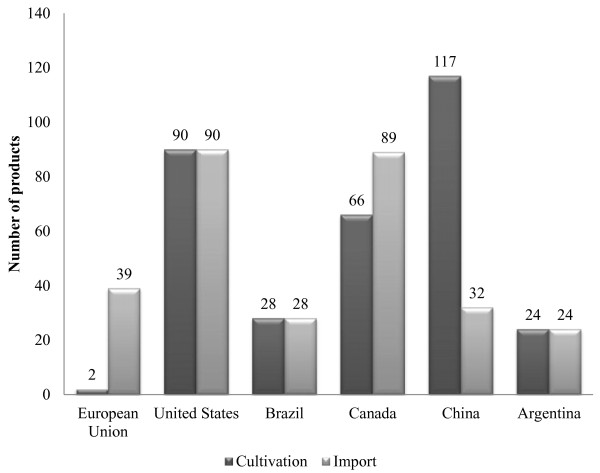
**Number of approved genetically engineered (GE) products in the EU, USA, Brazil, Canada, China and Argentina.** Data compiled from [123,126,127].

Animal agriculture is highly dependent upon GE crops. Table 3 shows the importance of GE crops to the animal feed export market. This creates a problem when there are “asynchronous approvals” of GE events, where an event is fully approved for commercial use in food and feed in one country, but not in others (Figure 3). This is particularly true for trade with the European Union (EU), as it has been estimated that 98% of soybean meal and 80% of all animal feed consumed in the EU is imported, of which more than half is from GE crops imported from Brazil, the USA, and Argentina [123]. The EU imports approximately 70% of the soybean meal used in animal feed and of this 80% is GE [124]. The proportion of GE in animal feed is likely even higher in the US where 93% of soy and 88% of all corn grown were GE varieties in 2012 [1].

**Table 3:** **Share of global crop trade accounted for by GE crops in 2011/12 (million tonnes)**[125]

	**Soybeans**	**Maize (Corn)**	**Cotton**	**Canola**
Global production	238	883.5	27.0	61.6
Global trade (exports)	90.4	103.4	10.0	13.0
Share of global trade from GE producers	88.6 (98%)	70.0 (67.7%)	7.15 (71.5%)	9.9 (76%)
Estimated size of market requiring certified conventional (in countries that have import requirements)	3.0	4.4	Negligible	Negligible
Estimated share of global trade that may contain GE (i.e., not required to be segregated)	87.4	70.0	71.5	9.9
Share of global trade that may be GE	96.7%	67.7%	71.5%	76%

Notes: Estimated size of market requiring certified conventional in countries with import requirements excludes countries with markets for certified conventional for which all requirements are satisfied by domestic production (e.g. maize in the EU). Estimated size of certified conventional market for soybeans (based primarily on demand for derivatives used mostly in the food industry): EU 2.0 million tonnes bean equivalents, Japan and South Korea 1 million tonnes.

The EU does not provide for any tolerance threshold for the accidental presence of unapproved GE events that have received regulatory approval in other countries. A 0.1% “technical solution” threshold was approved for feed material authorized in a non-EU country and for which an EU authorization request for the GE event in question has been lodged with EFSA for at least 3 mo or for which the authorization has expired. The 0.1% threshold is considered to be commercially unviable [124], and as more GE crops are grown in major grain exporting countries there is a very real possibility of major trade disruptions resulting from asynchronous approvals. Livestock production accounts for 40% of the total value of agricultural production in Europe. It has been estimated that if the EU were not able to import soybean protein from outside the EU it would only be able to replace 10-20% of imports by high protein feed substitutes, and that this would result in a substantial reduction in animal protein production, exports and consumption, and a very significant increase in animal protein imports into the EU [128].

### Cost:benefit analysis

In the abstract, the best approach for the regulatory evaluation of GE crops is one that allows new GE crops to be commercialized, while preventing new risks to animal and human health and the environment [129]. It is almost certain that animal agriculture globally will continue to rely on feed from GE crops. To date, commercialization of GE crop varieties has been associated with disproportionately high regulatory costs, regulatory delay, and considerable trade uncertainty. This has made their commercialization prohibitively expensive for all but the largest, multinational corporations.

Given the weight of scientific evidence on the safety of GE crops and the considerable expense involved, the decision to conduct an animal feeding study with a GE crop should be based on the need to answer a scientific question that cannot be addressed using *in silico* and *in vitro* methods. A reasonable hypothesis-driven food safety concern should be the driver for the additional expense and use of experimental animals required for such studies. The specific objectives and the rationale for choosing to perform a long-term chronic toxicity and/or carcinogenicity study should be clearly documented before conducting the experiment based on a remaining unanswered safety question following a 90-d rodent feeding trial.

Mandating long-term or target species animal feeding studies costing millions of dollars based on the process used to make a GE crop, rather than the unique traits and/or phenotype associated with the gene/crop combination is not justified based on the weight of evidence. Regulations triggered by how products are made are inconsistent with science-based risk assessment unless there is something inherently risky about the process, as compared to existing methods. A substantial body of evidence shows that GE crops are no more risky than conventionally bred crops, and mandating costly animal feeding studies in the absence of a reasonable unaddressed food safety concern associated with the novel trait and/or phenotype cannot be scientifically justified and overregulation is an indulgence that global food security can ill afford.

Moreover, the current approach to regulating GE crops does not evaluate the potential benefits that might be associated with the introduction of a GE crop. There have been substantial economic, production, and environmental benefits associated with the introduction of the first generation of GE crops [122,129-132]. All technologies are associated with both risks and benefits, and few would be adopted in the face of a risk-only analysis. In some cases GE crops may pose fewer risks than are implied by the non-GE alternative (e.g. reduced mycotoxin in *Bacillus thuringiensis* corn [107]). Perhaps as importantly, the lives saved or other benefits derived from risk assessment and management must be large enough to offset the costs and deferred potential benefits. The poorest and most vulnerable disproportionately bear the costs and impacts of excess regulation [129].

At the current time there are no international standards for assessing the potential benefits associated with the release of a new GE organism, although in many countries there are increasing calls for a risk-benefit analysis to form an integral part of GE regulatory frameworks [133]. Shifting from a risk-only regulatory focus to one that includes a risk:benefit analysis would enable a more balanced and harmonized evaluation of the likely impacts of introducing a new GE organism.

## Conclusions

Hundreds of peer-reviewed animal feeding studies have repeatedly shown that GE plants can safely be used in feed, and rDNA fragments have never been detected in products (e.g. milk, meat, eggs) derived from animals that consumed GE feed. Given the 15 yr history of safe use and absence of scientific evidence to suggest GE is associated with unique risks, whole food/feed animal feeding studies on GE crops should be reserved for GE crops where the novel phenotype results in a reasonable food safety concern that remains unanswered following all other analyses. Indiscriminately requiring long-term and target animal feeding studies based on a GE process-based trigger is not scientifically justified and will have an inhibitory effect on the development and commercialization of potentially beneficial GE feed crops in the future. World-wide GE regulations have disproportionately focused only on the potential risks associated with GE technology and commercialization of GE crops has been associated with a high regulatory compliance expense which has slowed adoption, particularly in small and poor developing countries. It is time for regulatory frameworks to consider the benefits in addition to any unique risks associated with GE technology. There are many current (increased yields, reduced insecticide use, improved feed quality), and potential future benefits of GE including feed crops with enhanced nutritional characteristics and durability. Regulatory frameworks should formally evaluate the reasonable and unique risks and benefits associated with the use of both GE plants and animals in agricultural systems, and weigh them against those associated with existing systems, and the opportunity costs associated with regulatory inaction.

## Abbreviations

GMO: Genetically modified organism; GE: Genetically engineered; OECD: Organisation for economic co-operation and development; GNA: *Galanthus nivalis* agglutinin; EFSA: European food safety authority; FDA: Food and drug administration; mRNA: messenger RNA (ribonucleic acid); DNA: deoxyribonucleic acid; EU: European union; rDNA: recombinant DNA.

## Competing interests

The author declares no competing interests.

## Author’s contributions

AVE drafted and approved the final manuscript.

## Author’s information

AVE is an Animal Genomics and Biotechnology Extension Specialist in the Department of Animal Science at the University of California, Davis where she received a Ph.D. in Genetics. Her extension program provides research and education on the use of animal genomics and biotechnology in livestock production systems.
